# Serum Therapy*Read at Palestine meeting East Texas Medico-Chirurgical Society, May, 1902.

**Published:** 1902-10

**Authors:** H. N. Graves

**Affiliations:** Georgetown, Texas


### TEXAS MEDICAL JOURNAL.

ESTABLISHED JULY, 1885.

PUBLISHED MONTHLY.—SUBSCRIPTION $1.00 A YEAR.

VOL. XVIII. AUSTIN, OCTOBER, 1902. No. 4.

### Original Contributions.

For Texas Medical Journal.

### Serum Therapy.*

BY H. N. GRAVES, M. D., GEORGETOWN, TEXAS.

Serum therapy is the crowning glory of the therapeutics of the nineteenth century, and the story of its development will prove always one of the most, if not the most, interesting chapters in medical history. And the human race will perhaps owe to such men as Jenner, Koch, Pasteur,—to the latter we doubtless owe most,—Behring, Roux, Kitasato, Salmon, Smith, Ehrlich, and others, our greatest debt of gratitude for all ages to come, for they have discovered a new world, and placed the study of medicine among the exact sciences.

When we study the history of "serum therapy" we are much surprised at the tardiness, if not timidity, in this investigation, since for several thousand years the snake charmers of India practiced the principle of "immunization" by using small and increasing doses of the venom of reptiles, nor did the profession until recently fully comprehend the scope of the Jennerian discovery. It remained for Koch and Pasteur to catch the great thought, the great principle of "immunizing," notwithstanding we knew for ages that many diseases left their immunity in the system.

"Serum therapy" is based on the fact that infectious diseases are caused by germs, both animal and vegetable, many of them isolated

*Read at Palestine meeting East Texas Medico-Chirurgical Society, May, 1902.

and recognized, and when our glasses are more powerful we will doubtless be able to identify them all.

Serums are divided into two classes, antitoxic and infectious. That is to say, some germs, such as the diphtheritic, produce a toxin which does the fatal work, the germ itself being harmless, but the product of the germ is the poison, like the snake whose flesh is edible but whose venom is fatal; and another class of germs which produce no toxic body, but it seems the germ *per se* causes the fatality—as the streptococcic. Since the first class produce a toxin we have for them an antitoxin, or anti-body, as the second class make no toxin, we can have for them no antitoxin, but a simple serum, such is the anti-streptococcic serum, more in the nature of a vaccine, a better prophylactic than curative, yet by far the better practice in the streptococcic diseases, such for instance as puerperal fever, erysipelas and pyemia. Marmorek's serum is made from a mixture of the cultures of several of these germs.

From what is known now of germ diseases and serum treatment it can be but a short while until the curative serum will be discovered for all germ-produced diseases. In the near future we are led to expect a specific serum for at least typhoid, pneumonia, and scarlatina, in fact, recently Professor von Leyden, of Berlin, concluded that though the scarlatinal germ has so far eluded the present microscope, yet the blood of patients suffering from scarlatinal infection might carry the antitoxin and procured blood from these convalescents, with the serum from which he has treated quite a number of scarlet fever patients with very gratifying results, but a great commercial difficulty arises with this serum, since it is only procurable from the human subject. Yet he may have discovered a great principle.

With what facts that have now been gleaned by the patient toiler with his peering glass along merely the shores of these great seas of knowledge, yet to be sounded, the scientist should count himself happy that he was born today, nor deem it a little or lowly thing to gather great life-saving knowledge from so small things as mosquitoes and micro-organisms, and while perforce we say "all hail" to the plodding, patient students who dug deep down amid the unseen and unknown of God's infinitesimal creation and gave to humanity in "serum therapy" the greatest boon which has come to it since Adam was a boy, yet there remains more, much more for the student-philanthropist to do along these lines. How much the more worthy the man, and the more to be honored and loved, who converts the very corpuscles of his own blood into thought power, and wading deep down into the plasmodium of disease, there seeking and finding one, if only one little death-dealing micro-organism *and its antidote,* thereby striking off one from death's long list, than the man with his airship, or his submarine boat, or his wireless telegraphy. Who of us with a heart larger than a microbe's had not the rather restore a sick baby to its heart-breaking mother than to talk across the sea, or wear the crown of Edison? And that's just what serum therapy does, towit, anti-diphtheritic serum has reduced the dreadful ravages of that disease from forty-seven per cent. to less than seven, and is a certain prophylactic.

Why is not anti-tetanic serum as much a specific as anti-diphtheritic, since both are antitoxins? Both are theoretically the same, but in our clinical experience we get less satisfactory results from the anti-tetanic serum, because we do not introduce it into the economy soon enough, for in our laboratory work we inject both the toxin and the antitoxin at the same time, with almost certain favorable results, while in general practice we do not use the antitoxin until we see the symptoms, rigidity of muscles, etc., at which period the germs have already thrown out more toxin than the antitoxin can overcome, have already destroyed some essential center of the brain or cord. If we could know very early that the tetanus germ was introduced and exhibit the antitoxin, the patient would probably recover; you will remember that the tetanus toxin kills in smaller doses than any other known poison. The anti-tetanic serum is a safe prophylactic; Nocard proved it.

What is the *modus operandi* of the antitoxins and other serums in overcoming the toxins or germs in the human economy? There is little known absolutely as to their action, but several theories account fairly well for this action. The phagocites, with their leucocytes, seem to stand always ready at the door of nature's mother-cells of our systems to repel, defend and defeat all harmful invasions by a repelling or combining force, maybe chemical, neutralizing the toxins. This phagocytosis has doubtless much to do with protecting us from these poisons. Ehrlich suggests his "side chain theory," which holds there are bodies already existing in the blood which are receptors or side chains; then there are bodies built up in the blood by immunization on animals; these are intermediary bodies; there are also bodies called complements. When the toxins enter the body, these receptors, which are present, will grasp or combine chemically with a certain amount of the toxin and neutralize it, the intermediary body with its complement doing the same. Since the intermediary body is the product of immunization, it can be built up by injecting antitoxin, so this body with its complement will neutralize all the toxin it can combine with. In the case of germs the theory bacteriolysius plays a part, where germs enter the body they are agglutinized, this lytic or dissolving principle is built up by a process of immunization, animals immunized against the germ are able to dissolve the germ, this lytic principle produced by immunization is the intermediary body—antitoxin is an intermediary body—and is able to build up receptors in the body, which are able to defend the body against toxins or germs.

As to the manufacture of the serums I can fairly illustrate them all by describing the processes (laboratory) of one, and as the antidiphtheritic is the one most commonly used by the practitioner, I will take it for my example. Now, to get an antitoxin and its value you first must procure the toxin and establish its value. We get a specimen of membrane from a throat affected with diphtheria, verified by all tests; securing a few germs from this they are placed in a culture media, bouillon (beef tea) or agar (gelatine) where they colonize, all other germs being eliminated by processes of dilution. After some days of development we have our toxin, the more virulent the better, then carbolic or tridesol is added to kill the germs, then filtration. The germ itself is not injected. The diphtheritic germ, *per se,* is non-toxic, nor do the animals injected with the toxin have typical diphtheria, the toxin acting only as a general constitutional poison, producing no membrane or special throat symptoms. To establish the value of each preparation of toxin we take a series of guinea pigs, usually five, weighing practically the same, and number them and mark them with red, yellow, etc., paints, to distinguish them. Pig No. 1 is given, say, 1-200 of a cc., pig No. 2 is given a little more, and so on to pig No. 5, which gets, say, 1-100 of a cc. of the toxin. Time element is a factor in establishing an accurate value, so at the expiration of twenty-four hours if we find pigs Nos. 1 and 2 still living we did not find the unknown quality we were seeking, but if pig No. 3 *is dead,* we get in it our value; say the amount given it was 1-150 cc., then multiplying relative weight of pig to horse's weight we get a definite dose of the toxin. The next step is work with the horses, and you must understand everything from start to finish is under the strictest and most aseptic and antiseptic conditions, from hot air blast to cold storage, all modern inventions for cleanliness are employed. The horse is injected, hypodermic, with his initial dose of toxin, about 1-15 cc. (one drop) and gradually increased as they can bear it, up to as high as possible, some tolerating comparatively little, others reaching as high a tolerance as

1000 ccs. three times a week. The horse is usually treated from three to six months before his serum is valuable, then he is bled from the jugular vein to about three gallons, received in suitable containers of one quart. These containers are kept in cold storage of an even temperature for several days, when the serum, which has separated, is decanted off and tested bacteriologically and microscopically for foreign germs. This blood yields about fifty per cent. serum. This serum is *antitoxin;* it is taken to the pigs' room and another same number of pigs, weighing practically the same as before, is taken and each pig is given (hypodermic) *ten* times the minimal fatal dose of the toxin, which in the illustration used above was 1-150 cc., and at once each pig receiving this ten-minimal fatal doses will receive a certain amount of the antitoxin, varying from 1-5000 to 1-2000 ccs., then at the expiration of twenty-four hours the pig which is *still living* and received the smallest amount of antitoxin gives us the value of the serum. Then we multiply this minimal dose of antitoxin, which saved the life of the pig, by ten and call it a unit. The formula reads: The unit of antitoxin is one hundred times the smallest amount of antitoxin that will save the life of a guinea pig from the smallest fatal dose of the toxin.

A few words as to the administration of anti-diphtheritic serum. Since it is absolutely non-toxic in any quantity (Dr. Campbell White, of Baltimore, having exhibited 110,000 units with no untoward symptoms), the dose should be large enough to slough the membrane, nor is the interval between doses so long as formerly, six to eight hours being the average time between doses with doctors I see. I have a friend who uses it every two hours. The rule of practice is to exhibit in merely suspicious cases, verifying diagnosis later. The basis dosage for a child two years with laryngeal diphtheria in early stage or mild case, 2000 units and repeat in six or eight hours if no improvement. The prophylactic dose for all ages is 500 units and protects from three to four weeks. A point to remember is that the antitoxin does not destroy the diphtheritic germ, only neutralizes its toxin, so the more care should be exercised in the quarantine of convalescents. Antitoxin sometimes produces pain in joints and a rash; they are temporary and require no treatment.

Antitoxin, given early, seems to prevent the sequelæ, such as paralysis, heart failure, etc., but if administered late in the disease, may not influence the after troubles. It is oftener now injected into the cellular tissues than intra-muscular.

The following table, with which I close, is very significant:

Mortality rate in diphtheria, where antitoxin was used on first and second days of disease, six per cent.; where used on third day of disease, twelve per cent.; where used on fourth day of disease, twenty-three per cent.; where used on fifth day and later, thirty-five per cent.

